# Retrospective Analysis of the Areas Responsible for Light Flash and Odor During Proton Beam Therapy and Photon Therapy

**DOI:** 10.7759/cureus.44790

**Published:** 2023-09-06

**Authors:** Yoshiko Oshiro, Masashi Mizumoto, Toshio Miyamoto, Taisuke Sumiya, Den Fujioka, Haruka Shirataki, Masatoshi Nakamura, Toshiki Ishida, Takashi Iizumi, Takashi Saito, Haruko Numajiri, Hirokazu Makishima, Kei Nakai, Kazushi Maruo, Takeji Sakae, Hideyuki Sakurai

**Affiliations:** 1 Department of Radiation Oncology, Tsukuba Medical Center Hospital, Tsukuba, JPN; 2 Department of Radiation Oncology, University of Tsukuba Hospital, Tsukuba, JPN; 3 Department of Radiation Oncology, Proton Medical Research Center, University of Tsukuba Hospital, Tsukuba, JPN; 4 Department of Radiation Oncology, Ibaraki Prefectual Central Hospital, Tsukuba, JPN; 5 Department of Radiation Oncology, University of Tsukuba, Tsukuba, JPN; 6 Department of Biostatistics, Institute of Medicine, University of Tsukuba, Tsukuba, JPN

**Keywords:** proton, brain, radiotherapy, odor, light flash

## Abstract

Background

Abnormal sensations were frequently experienced by patients who received irradiation of the brain or head and neck region. We have previously suggested correlations with irradiation of the nasal cavity and retina.

Purpose

We performed a retrospective dose-volume histogram analysis focused on the brain and head and neck tumor to examine the relationship between these abnormal sensations and the details of irradiation.

Methods

Multivariate logistic regression models were applied for the presence or absence of light flash and odor. Gender, age, radiotherapy method (proton beam therapy vs. photon radiotherapy), dose of retina, optic nerve, chiasmatic gland, pituitary, nasal cavity, oral cavity, frontal lobe, parietal lobe, occipital lobe, temporal lobe, amygdala, and hippocampus were set as candidates of explanatory variables.

Results

Light flash and odor during radiotherapy have been suggested to be associated with younger age and retina and nasal cavity irradiation. Multivariate analyses including dose-volume histograms indicated that light flash was related to age, chiasmatic gland irradiation, and pituitary dose, and odor was related to age and nasal cavity irradiation.

Conclusion

Our results indicate that light flash during radiotherapy is caused by irradiation of the visual pathway and that odor is caused by irradiation of the nasal cavity or olfactory bulb.

## Introduction

Some patients complain of odor or light flash during radiation therapy (RT). Previous studies have suggested that irradiation of the nasal mucosa and ozone generation are responsible for the odor during RT, and that light flash is mainly caused by a Cherenkov effect, a phenomenon in which light is emitted when charged particles pass through a medium faster than the speed of light, in the context of exposure of the retina or vitreous of the eyes [1-4]. However, Kosugi et al. reported a patient who sensed odor during RT after resection of the olfactory epithelium due to olfactory neuroblastoma [5]. Based on the prospective studies of odors and light flash during photon RT and proton beam therapy (PBT), we have previously suggested correlations with irradiation of the nasal cavity and retina [6-9]. These abnormal sensations were frequently experienced by patients who received irradiation of the brain or head and neck region. However, some patients who underwent body trunk irradiation also complained of odor or light flash [6-9]. These events are sometimes experienced clinically, but few reports have been scientifically considered. Therefore, in the current study, we performed an additional dose-volume histogram analysis of two previously published prospective studies [6,9] focused on the brain and head and neck tumor to examine the relationship between these abnormal sensations and the details of irradiation. This article was previously posted to the medRxiv preprint server on January 27, 2023 [https://www.researchsquare.com/article/rs-2508561/v1?].

## Materials and methods

A prospective observational study was performed in all adult patients (≥20 years old) who received photon RT or PBT to the brain or head and neck area at two centers between January 2019 and August 2020, using a previously described survey [6-9]. A retrospective analysis of the relationships of flashes and odor with irradiated sites, including the nasal cavity, oral cavity, optic nerve, chiasmatic gland, retina, frontal lobe, parietal lobe, temporal lobe, occipital lobe, hippocampus, amygdala, cerebellum, and brainstem, was then performed. The nasal cavity and olfactory bulb could not be separated due to their proximity and were included in the irradiation field together. Irradiated means a site receiving 10% or more of the prescribed dose in this study.

Statistics

Multivariate logistic regression models were applied for the presence or absence of light flash and odor. Gender, age, RT method (PBT vs photon RT), dose of retina, optic nerve, chiasmatic gland, pituitary, nasal cavity, oral cavity, frontal lobe, parietal lobe, occipital lobe, temporal lobe, amygdala, and hippocampus were set as candidates of explanatory variables. Age was analyzed as a continuous variable in the multivariate analysis. Variable selection was performed using a stepwise method with a criterion of p <0.10. The c statistic for each selected model was calculated with 10-fold cross-validation. Statistical significance was defined as p <0.05. All statistical analyses were conducted with R software ver. 4.2.1 (R Core Team, Vienna, Austria).

## Results

The retrospective analysis was performed in 162 of 621 patients who received photon RT or PBT for a head and neck or brain tumor. The median age was 67 (20-90) years. Of the 162 patients, 59 (36.4%) experienced a light flash and 28 (17.2%) sensed an odor during RT. The details of light flash and odor are shown for each factor in Table 1.

**Table 1:** Presence of light flash and odor.

Characteristics	Light flash (n=59)	Odor (n=28)
Age		
≤67 years	38/79 (48%)	21/79 (27%)
>67 years	21/82 (26%)	7/82 (9%)
Gender		
Male	41/101 (41%)	22/101 (22%)
Female	18/60 (30%)	6/60 (10%)
Radiotherapy technique		
Photon radiotherapy	44/120 (37%)	20/120 (17%)
Proton beam therapy	15/41 (37%)	8/41 (20%)
Retina		
Irradiated	55/97 (57%)	24/97 (25%)
Not irradiated	4/64 (6%)	4/64 (6%)
Optic nerve		
Irradiated	54/99 (55%)	24/99 (24%)
Not irradiated	5/62 (8%)	4/62 (6%)
Chiasmatic gland		
Irradiated	51/96 (53%)	23/96 (24%)
Not irradiated	8/65 (12%)	5/65 (8%)
Pituitary		
Irradiated	52/97 (54%)	23/97 (24%)
Not irradiated	7/64 (11%)	5/64 (8%)
Nasal cavity		
Irradiated	54/103 (52%)	26/103 (25%)
Not irradiated	5/58 (9%)	2/58 (3%)
Oral cavity		
Irradiated	32/76 (42%)	13/76 (17%)
Not irradiated	27/85 (32%)	15/85 (18%)
Frontal lobe		
Irradiated	53/106 (50%)	22/106 (21%)
Not irradiated	6/55 (11%)	6/55 (11%)
Parietal lobe		
Irradiated	40/92 (43%)	18/92 (20%)
Not irradiated	19/69 (28%)	10/69 (14%)
Occipital lobe		
Irradiated	40/93 (43%)	16/93 (17%)
Not irradiated	19/68 (28%)	12/68 (18%)
Temporal lobe		
Irradiated	45/103 (44%)	19/103 (18%)
Not irradiated	14/58 (24%)	9/58 (16%)
Amygdala		
Irradiated	46/99 (46%)	20/99 (20%)
Not irradiated	13/62 (21%)	8/62 (13%)
Hippocampus		
Irradiated	45/101 (45%)	20/101 (20%)
Not irradiated	14/60 (23%)	8/60 (13%)

For light flash, age (p=0.04), chiasmatic gland irradiation (p=0.015), and dose to the pituitary (p=0.02) were significant factors in multivariate analysis, but the retina was not a significant factor (p=0.058) (Table 2).

**Table 2:** Multivariable logistic regression analysis of the presence of light flash. Statistical significance was defined as p <0.05. CI, confidence interval.

Variable	Odds ratio	95% CI	p-Value
Age	0.968	0.938-0.998	0.040
Retina	1.020	1.000-1.043	0.058
Nasal cavity	1.015	0.998-1.032	0.088
Oral cavity	0.989	0.977-1.000	0.059
Chiasmatic gland	0.928	0.866-0.977	0.015
Pituitary	1.078	1.020-1.159	0.020

Of the 59 patients who saw a light flash, the retina, chiasmatic gland, or optic nerve was not included in the irradiated field in four, eight, and five cases, respectively; however, at least one of the retina, optic nerve, chiasmatic gland, and occipital lobe, all of which are involved in vision, was included in the irradiated area in these patients (Table 3).

**Table 3:** Irradiation doses in patients who experienced light flash, but in whom the retina, chiasm, or optic nerve was not included in the irradiated field.

Age	Retina	Chiasm	Optic nerve	Pituitary	Frontal	Parietal	Temporal	Occipital	Amygdala	Hippocampus
66	0	10	30	20	80	0	0	0	0	0
56	0	10	10	10	10	0	10	0	10	10
59	0	0	0	0	0	100	0	90	0	0
61	0	0	0	0	0	0	20	100	0	0
55	20	0	0	0	30	100	40	90	30	30
61	100	0	0	0	0	0	0	0	0	0
69	60	0	0	0	0	0	0	0	0	0
44	100	0	100	20	90	0	0	0	0	0
72	100	0	90	0	90	0	0	0	0	0
68	90	0	60	50	90	0	0	0	0	0

For odor, younger age (p=0.004) and irradiation of the nasal cavity (p=0.003) were significant factors in the multivariate analysis (Table 4).

**Table 4:** Multivariable logistic regression analysis of the presence of odor. Statistical significance was defined as p <0.05. CI, confidence interval.

Variable	Odds ratio	95% CI	p-Value
Gender (female/male)	0.355	0.116-0.955	0.051
Age	0.950	0.916-0.982	0.004
Nasal cavity	1.020	1.008-1.035	0.003

Only one patient sensed an odor without the nasal cavity being included in the irradiation field. Tables 2, 4 show the result of multivariate analysis. The c statistics for models of light flash and odor were estimated as 0.824 and 0.802, respectively.

## Discussion

A light flash is thought to be due to Cherenkov radiation and previous studies have suggested that this is triggered by irradiation of the retina [6-8]. The current study suggested that the chiasmatic gland is a significant factor for light sensation and that retina irradiation tends to be related to light sensation, although the results did not suggest significance. Also, the occipital lobe was irradiated in two patients without irradiation of the retina, optic nerve, or chiasmatic gland.

The retina contains light-sensitive cells referred to as cones and rods, which detect color and light intensity, respectively. The light and color information caught by these cells is translated into digital signals that are transmitted to the optic nerve, through the lateral lemniscus, and finally to the visual cortex located in the occipital lobe, where it is used to form a combined light and color image. The visual information then splits into ventral and dorsal visual pathways. Thus, our results suggest that irradiation of the visual pathway, including the retina, optic nerve, chiasmatic gland, and occipital lobe, leads to a light sensation.

Regarding odor, all patients except one who felt odor received irradiation to the nasal cavity. The odor of irradiation is often described as that of ozone [1-3,7-9]. Ozone is formed in the vicinity of electron beams by collisions between high-energy electrons and oxygen molecules. However, ozone is unstable and would only be detectable at low concentrations if produced adjacent to the olfactory receptor region [6]. An unpleasant odorous sensation may also be due to free radicals produced by radiation that remove electrons from stable molecules in the olfactory membrane or the overlying mucous layer in the nasal cavity [6].

Our previous results suggested that patients who received RT for the body trunk could sense odor [6-9]. Kosugi et al. presented the case of a patient who experienced odor during tomotherapy after resection of the olfactory epithelium [5], and it is also well known that a foul odor is caused by uncinate epilepsy. The olfactory pathway starts from the olfactory epithelium, and then axons of the olfactory nerve project through the olfactory bulb and accessory olfactory bulb to the piriform cortex and amygdala. The foul odor during uncinate epilepsy is initiated by a hook located in the limbic cortex, which is involved in olfactory function. Thus, it is possible that odor may be sensed by irradiation of the olfactory pathway, but this occurs less frequently than sensing of light due to irradiation of the visual pathway.

The presence of secondary electrons produced during RT may provide another explanation for sensing light and odor in patients whose nasal passages are not irradiated. These electrons are X-rays that have higher energy and a greater spatial distribution than that of proton beams. Therefore, the electrons may act on the nasal cavity and retina. In fact, a previous study suggested that some patients who received RT to the trunk felt light flash and odor, and most of these patients received photon RT. Secondary electrons have a range of a few centimeters and it is possible that these electrons may hit the retina, olfactory membrane, or olfactory bulb. We also note that RT is essential for the treatment of pediatric tumors [10] and our results indicate that children are more likely to sense light flashes and odors. Therefore, it should be kept in mind that the inclusion of the visual pathway or nasal cavity in the irradiation range in pediatric cases makes it likely that the patient will sense light and odor. Sasai et al. review on odors during RT [11]. Due to the paucity of articles on odor during RT, only 12 articles were included in the review, including case reports. The causes of odor during RT remain unknown, as there are no reports with a high level of evidence and patient backgrounds vary. However, similar to our analysis, Sasai et al. conclude that odor is more likely to be perceived if olfactory regions are included in the irradiated area. Mai et al. conducted a prospective study on abnormal visual and olfactory sensations during RT [12]. This prospective study provides a detailed analysis of the background to the perception of abnormal light and odor during RT. The results of the study were similar to our previous analyses, with irradiation of the skull base and brain and younger age being significantly associated with the perception of abnormal sensations. However, limitations of the present study include the fact that the evaluation of abnormal sensations is derived from individual sensations and is not objective and that no evaluation of irradiation volume has been carried out regarding RT. Additional validation is being carried out in a new prospective study to assess abnormal sensations during irradiation.

## Conclusions

In conclusion, there are still unknown aspects of the abnormal sensations experienced during irradiation. However, our results indicate that light flash during RT is caused by irradiation of the visual pathway and that odor is caused by irradiation of the nasal cavity or olfactory bulb.
